# Estimates of Water Consumption of Growing Pigs under Practical Conditions Based on Climate and Performance Data

**DOI:** 10.3390/ani13091547

**Published:** 2023-05-05

**Authors:** Patrick Schale, Engel F. Arkenau, Armin O. Schmitt, Sven Dänicke, Jeannette Kluess, Angelika Grümpel-Schlüter

**Affiliations:** 1 Johann Heinrich von Thünen-Institute, Federal Research Institute for Rural Areas, Forestry and Fisheries, Institute of Agricultural Technology, Bundesallee 47, 38116 Braunschweig, Germany; 2 Federal Ministry of Food and Agriculture, Directorate 82 Digital Innovation, Wilhelmstraße 54, 10117 Berlin, Germany; 3 Breeding Informatics, Department of Animal Sciences, Georg-August-Universität Göttingen, Margarethe von Wrangell-Weg 7, 37075 Göttingen, Germany; 4 Friedrich-Loeffler-Institut, Institute of Animal Nutrition, Bundesallee 37, 38116 Braunschweig, Germany

**Keywords:** fattening pigs, water consumption, feed, temperature-humidity index, THI, climate

## Abstract

**Simple Summary:**

Meeting the water requirements of fattening pigs is crucial for animal welfare in successful animal husbandry. At the same time, barn climate has a significant influence on the water requirements of fattening pigs. Equations for the estimation of water consumption are useful in this context. However, it is questionable whether existing equations match modern pig genetics and housing conditions. Therefore, the aim of this study was to evaluate the water consumption of modern fattening pigs and compare the measured values with the predicted values using equations from the literature. It was found that the measured water consumption was, in most cases, higher than the calculated one. As a result, six new equations for the prediction of water consumption were derived and discussed. More data are needed to further specify these new equations.

**Abstract:**

The water consumption of fattening pigs was recorded under practical conditions and compared with calculated water consumption. The experiment was carried out in the summer of 2020 with 79 fattening pigs. Data loggers were used to record the climate data, such as temperature and relative humidity. These data were used to calculate the temperature-humidity index (THI). It was found that there were sometimes considerable discrepancies between the measured and the calculated water consumption. One possible reason for this discrepancy could be the age of the existing water requirement equations, as in recent decades there has been a clear breeding development and thus a strong increase in pig performance. Based on these deviations, six new water consumption equations were established, which considered the variables body weight (BW), temperature, THI and feed consumption. It was found that the THI and BW should be included in one equation as predictor variables and the evaluation also showed good results. Its use, in practice, should also be considered. Overall, it became apparent that there is still a need for further research to make water consumption equations more precise. This would require a larger database.

## 1. Introduction

Feeding, water supply and housing climate are important factors for successful fattening pig husbandry. An insufficient water supply leads to a decrease in feed intake and, as a result, performance and feed efficiency decline [1]. 

This shows how important a sufficient water supply is both in terms of animal welfare and from an economic point of view. At the same time, the barn climate also has a significant influence on water intake [2]. When the air temperature increases, the water consumption of pigs also increases [3,4]. In the course of climate change, the importance of an adequate water supply will continue to increase due to more frequent extreme weather events, such as hot spells. Thus, in addition to the water supply, the barn climate has become an increasingly important factor for successful fattening pig husbandry. Here, temperature and relative humidity are of particular interest. Since pigs can sweat only to a very small extent, they achieve their thermoregulation by seeking cooling opportunities [5] or by panting [6]. This becomes more difficult the higher the relative humidity is at concurrently high temperatures [7,8]. Therefore, the possibility to easily check the barn climate is required. In cattle farming, the temperature-humidity index (THI) is often used for this purpose [9]. In pig farming, however, THI has rarely been used so far [10].

According to the Tierschutznutztierhaltungsverordnung (TierSchNutztV) [11], the animals must be provided with a sufficient quantity and quality of water. In order to estimate the water demand of fattening pigs, Schiavon and Emmans [12] and Gill [13] derived equations based on various variables, such as body weight (BW), feed consumption (FC) and ambient temperature. In light of the development of pig genetics over the last 30–50 years, the aim of this study was to verify these equations. At the same time, the THI was checked as a possible predictor variable. The experiment took place under practical conditions, as the aim was also to offer farmers a practical solution to estimate water consumption on their farms and then use this to control their livestock.

## 2. Animals, Materials and Methods

### 2.1. Experimental Setup and Data Collection

A total of 79 castrated male high-performing crossbred fattening pigs were randomly distributed to four pens (max. 20 animals/pen). The animals were housed at a stocking density of 0.75 m^2^/animal in the final fattening stage. The experimental house was heat-insulated and forced-ventilated with a fully slatted floor. At the start of the experiment, the piglets weighed 24.3 ± 4.3 kg on average across all pens. Data collection took place over 7 weeks and was divided into weeks 1–4 and 5–7. During the experiment, six pigs had to be removed from the pens or had to be written off as losses. The reasons for this were lameness, umbilical hernias and animal death.

The animals were also scored twice a week in terms of the animal welfare parameters of tail, ear and skin lesions and faecal contamination. Scores of 0 or 1 were given. A score of 0 corresponded to a positive evaluation, i.e., no injuries or faecal contamination. If lesions occurred, they were documented with a score of 1. Faecal contamination was also given a score of 1. Soiling was considered when it affected more than 10% of the body surface. The scoring scheme used was based on Schrader et al. [14] (Table 1). Every week, two people, each with experience in the field of animal welfare parameters, performed the assessment, resulting in one assessment per person and week. The assessments of the two persons were compared so that a good agreement in the assessment was achieved. The pigs already knew the two people from piglet rearing.

Once a week, the pigs were weighed individually, and the feed consumption was recorded by back weighing the residual feed quantities. All other data were recorded at the pen level. The weighing of the pigs took place in silence and in an environment familiar to the pigs.

The animals were offered feed for ad libitum consumption. The feed’s composition is shown in Table 2. When changing from experimental week 4 to 5, the feed from the former phase was blended over 5 days with the feed of the next phase.

Dry feeders were used at an animal-to-feeding space ratio of 4:1. In each pen two nipple drinkers were provided as double drinkers. The height of the drinkers was between 37.3 and 61.1 cm. At the beginning of the experiment, the flow rate of the drinkers was set at 0.8–1.2 L/min as recommended by Schrader et al. [14]. As the experiment took place under practical conditions, the watering flow rate was not adjusted later during the fattening period. The animal/drinker ratio was 10:1. It was ensured daily that the drinkers did not run when no animal was taking water in order to avoid distorted water consumption values. The daily water consumption was recorded using water meters. Each pen was equipped with a water meter JS Einstrahlzähler (Schlösser Armaturen GmbH & Co. KG, Olpe/Germany). Every morning, the water meters were read, and the water consumption was noted with the time. On days 43 and 44, there was a complete loss of data, so no values of water consumption were available for these days. On days 45 and 46, there was also a loss of data for one of the four experimental pens.

Since the experiment took place under practical conditions, only the consumption of feed and water, but not the intake, can be used in the following calculations. Losses, e.g., via manure, cannot be considered. 

In all compartments, a cooling system was installed, which was switched on twice a day, in the morning and in the evening, for 10 min via a timer. The cooling system was a garden house drip system, which was mounted under the ceiling and watered a part of the slatted floor. The barn was ventilated using negative pressure. The exhaust air was drawn out of the barn decentrally via fans. The supply air could be brought in from the outside, hereafter called supply air, or from the central corridor, hereafter called circulating air, as well as in mixed operation. The ventilation was controlled using a Fancom FSU.8 climate computer (Fancom BV, Panningen/Netherlands). During the experiment, ventilation was controlled via the curve mode (Appendix A, Table A1). A specific set of climatic conditions, e.g., to simulate heat phases, did not take place during the experiment, so the barn climate was determined purely by the weather.

The air temperature (T, °C), relative humidity (RH, %) and barometric pressure (hPa) were recorded every minute with data loggers HumiLog rugged, HumiLog rugged Plus and HumiBaroLog rugged Plus (measuring range temperature: −40–120 °C; measuring range relative humidity: 0–100%; barometric pressure: 10–1300 hPa) (Driesen + Kern GmbH, Bad Bramstedt/Germany). A data logger was placed centrally above each pen. In addition, another data logger was placed outside of the barn. This was located on a covered loading ramp to protect it from direct rain impact. The loading ramp was oriented southward. Direct sunlight and shading were alternated during the day. Another data logger was placed at the middle of the central corridor, which additionally recorded the barometric pressure. All data loggers were set at a recording interval of one minute. The data was read out using the software “InfraLog” (version 5.7.52 basic, Driesen + Kern GmbH, Bad Bramstedt/Germany).

Due to a malfunction of a data logger in the recording of the relative humidity in the animal area, the implausible values recorded for a short time were removed from the evaluation of three days.

Additionally, on the day of stabling and then every Tuesday, the concentrations of carbon dioxide (CO_2_), ammonia (NH_3_) and hydrogen sulphide (H_2_S) were measured in each pen at three defined measuring points distributed across the pen using the Honeywell Impact Pro noxious gas measuring device (measuring range: carbon dioxide (CO_2_): 0.2–2 Vol.-% (measurement accuracy: 0.1 Vol-%); ammonia (NH_3_): 0–100 ppm (measurement accuracy: 1 ppm); hydrogen sulphide (H_2_S): 0.4–50 ppm (measurement accuracy: 1 ppm) (Honeywell Deutschland Holding, Offenbach/Germany). For this purpose, measurements were taken in each pen at three defined measuring points distributed across the pen. Parallel to the pollutant gas measurement, the surface temperature of the slatted floor was also recorded at the three points using an IR-2200-50D USB infrared thermometer, measuring range −50 °C–2200 °C, resolution below 1000 °C at 0.1 °C, on the slatted floor (Voltcraft, Hirschau/Germany).

### 2.2. Data Analyses

As a first step, performance parameters, such as daily weight gain, feed and water consumption and feed conversion, were calculated.

In order to estimate the development of the animal welfare parameters, prevalence was calculated from the assessment results. For this purpose, the values for abnormal animals in each experimental week were added to each parameter. In the next step, prevalence was determined by calculating the percentage frequency of abnormal animals in relation to all animals assessed in the respective week. Furthermore, a cumulative score (CS) was calculated to record the development of the animal welfare parameters. This value was determined for each pig assessed. For this purpose, the individual scores for each animal-related parameter were added up and divided by the number of parameters. Thus, the CS could assume a minimum of 0.00 (all parameters normal) and a maximum of 1.00 (all parameters abnormal). In addition, the percentage frequency of negative deviation from the optimum (negative evaluation by the parameters during the assessment) was determined for each parameter. The mean values and standard deviations were then calculated from the individual values per animal.

Basically, all evaluations regarding water consumption were always related to the two phases, weeks 1–4 and weeks 5–7, and to the total fattening period. The animal weights, feed consumption quantities and temperatures were used to calculate the water consumption quantities. The animal weights were recorded once a week for each individual animal. For each pen, the average animal weight was then determined based on the individual animal weights. The average feed consumption per animal per day was calculated from the total amount of feed consumed per week. The recording of feed consumption was only possible once a week on the basis of the pen, as daily animal-specific data collection was not possible. Due to the six animal losses, feed consumption in the affected pens had to be evaluated by means of an average pen occupancy for the week in which the respective loss occurred. For this purpose, the mean value was calculated from the daily pen occupancy for the week and this value was then used as the number of animals for the entire week.

For the climate data values, the first step was to calculate the THI for each minute from the temperature (T, °C) and relative humidity (RH, %) data using the equation for the National Weather Service Central Region (NWSCR) [16]:

Equation (1):

(1) THI=[(1.8 * T)+32] − [0.55 * (RH/100)] * [((1.8 * T)+32)−58]

Subsequently, the weekly mean values of temperature, relative humidity and THI were determined.

The evaluation scheme of Eigenberg et al. [17] was used to assess the heat stress. For this purpose, the number of THI values calculated per minute was determined for each category of the evaluation scheme. Subsequently, the percentage distribution between the categories was computed.

The water consumption of the animals was calculated from the data recorded daily by the water meters. Since the water consumption was recorded at slightly different times, these were also recorded in order to be able to relate the water consumption quantities to 24 h within the framework of the evaluation.

The further evaluation of the water consumption was divided into a total of three evaluation procedures so that in the end the measured water consumption could be compared with the calculated water consumption on the basis of the following three units: L/pig and day, L/kg feed consumption and mL/kg BW.

Water consumption was calculated using the variables BW, feed consumption and temperature. The following equations from Schiavon and Emmans [12] were used for this calculation:

Equation (2) on the basis of the BW:

(2) $$\mathrm{water}\;\mathrm{consumption}\;[l/\mathrm{pig}\;d^{-1}]=0.076\;*\;\mathrm{BW}\;[\mathrm{kg}]+1.96$$

Equation (3) on the basis of the feed consumption:

(3) $$\mathrm{water}\;\mathrm{consumption}\;[l/\mathrm{pig}\;d^{-1}]=2.130\;*\;\mathrm{feed}\;\mathrm{consumption}\;[\mathrm{kg}/d]+1.57$$

Equation (4) on the basis of the environmental temperature:

(4) $$\mathrm{water}\;\mathrm{consumption}\;[l/\mathrm{pig}\;d^{-1}]=0.120\;*\;\mathrm{environmental}\;\mathrm{temperature}\;[{}^{\circ}C]+2.59$$

In addition, water consumption was calculated using the following two equations from Gill [13]:

Equation (5) on the basis of the BW:

(5) $$\mathrm{water}\;\mathrm{consumption}\;[l/\mathrm{pig}\;d^{-1}]=-1.42+0.25\;*\;\mathrm{BW}\;[\mathrm{kg}]\;-\;0.0021\;*\;\mathrm{BW}\;[\mathrm{kg}{]}^{2}$$

Equation (6) on the basis of the feed consumption:

(6) $$\mathrm{water}\;\mathrm{consumption}\;[l/\mathrm{pig}\;d^{-1}]=2.5+8.18\;*\;\mathrm{feed}\;\mathrm{consumption}\;[\mathrm{kg}/d]\;-\;1.978\;*\;\mathrm{feed}\;\mathrm{consumption}\;[\mathrm{kg}/d{]}^{2}$$

In the appendix, more information about the experimental conditions of Equations (2)–(6) can be found (Appendix A, Table A2 and Table A3).

#### Calculation of Water Consumption in L/Pig and Day, in Relation to Feed Consumption in L/kg FC and in Relation to Body Weight in mL/kg BW

**Figure A1:**
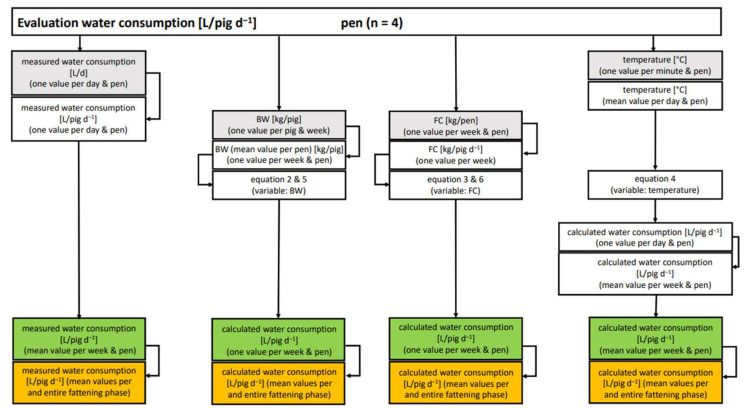
Data collection and data evaluation for the reference value L/pig and day: grey boxes = values measured in the pens; white boxes = calculated values; green boxes = calculated values used for statistical analysis; orange boxes = calculated values shown in the results; BW = body weight; FC = feed consumption.

**Figure A2:**
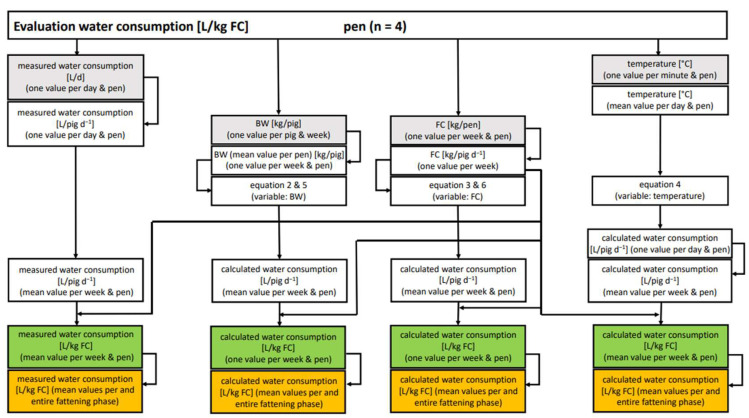
Data collection and data evaluation for the reference value L/kg FC: grey boxes = values measured in the pens; white boxes = calculated values; green boxes = calculated values used for statistical analysis; orange boxes = calculated values shown in the results; BW = body weight; FC = feed consumption.

**Figure A3:**
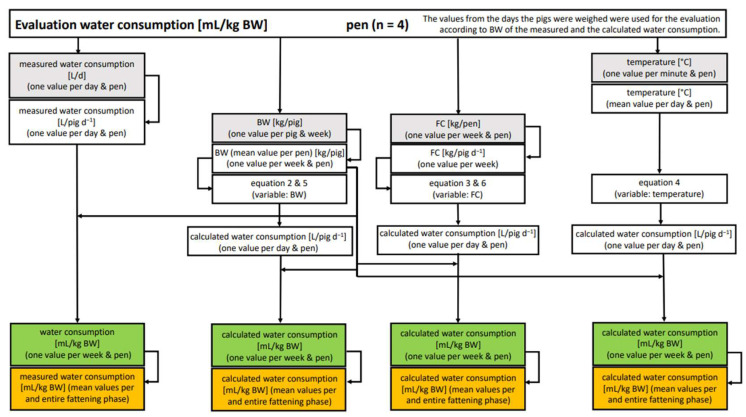
Data collection and data evaluation for the reference value mL/kg BW: grey boxes = values measured in the pens; white boxes = calculated values; green boxes = calculated values used for statistical analysis; orange boxes = calculated values shown in the results; BW = body weight; FC = feed consumption.

Water consumption was evaluated in the three units L/pig and day, L/kg FC and mL/kg BW. An average value was calculated for each fattening phase and for weeks 1–4 and 5–7. The calculated water consumption and the measured water consumption were compared (Appendix A, Figure A1, Figure A2 and Figure A3). In the last step, the percentage deviations between measured and calculated water consumption were calculated on the basis of the mean values for both the phases, weeks 1–4 and weeks 5–7, and finally for the entire fattening period. When calculating the percentage deviations, the calculated water consumption value was set to 100; therefore, if the measured water consumption was greater than the calculated water consumption, the calculated percentage deviation resulted in a positive value or a negative value in the opposite case.

For a regression between measured water consumption and feed consumption, weekly averages were also calculated over all 4 pens, so that one value per week was available for the two parameters. The basis here for the water consumption was L/pig and day and for the feed consumption was kg/pig and day.

### 2.3. Statistical Analyses 

The statistical programme SAS 9.4 (SAS Institute Inc., Cary, NC, USA, 2016) was used to calculate the correlation between feed and water consumption using the procedure “PROC CORR”.

Due to a partial lack of normal distribution, the Mann–Whitney U-test in SAS 9.4 was used to calculate significant differences between the measured and the calculated water consumptions for the two phases, weeks 1–4 and 5–7, and for the whole fattening period, calculated on the basis of L/pig and day, L/kg feed and mL/kg BW. Dunn’s test was used as a post hoc test and performed in the statistic programme R, version 4.2.2 (R Core Team, Vienna, Austria) employing the packages “dunn.test” (version 1.3.5) and “FSA” (version 0.9.3) [18]. The weekly mean values of the four pens were used in this calculation. Specified *p*-values of <0.05 were considered significant.

In total, six new equations were derived for the prediction of water consumption as the response variable. The statistical programme SAS 9.4 was again used for these regression analyses. The variables BW, feed consumption, temperature and THI were used as independent predictor variables. For doing so, data were prepared on a pen basis (n = 4) as follows:

- measured water consumption: average value for the entire week calculated from the daily consumption;
- BW: average weight of the animal group on the weighing day;
- THI NWSCR: mean value for the whole week calculated from the minute values;
- temperature: average value for the whole week calculated from the minute values;
- feed consumption: average feed consumption per pig and day calculated from the weekly feed consumption calculation.

Using the boot-strapping method, the response predictor variables were extrapolated to a data set of 5000 data and the correlations were calculated on the basis of these data. Specified *p*-values of <0.05 were considered significant. The regression was calculated throughout without an intercept since the water consumption was supposed to be explained by the predictor variables and a residual error.

## 3. Results

The performances of the animals are displayed in Table 3.

**Figure 1:**
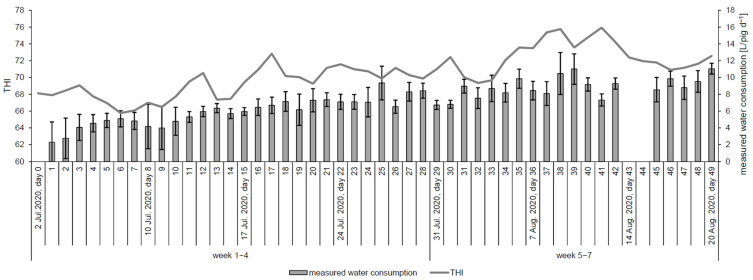
Measured water consumption [L/d] (mean ± sd) and development of THI (mean of the 4 pens) over the experimental period.

The prevalence of the different animal welfare parameters and CS showed a fluctuating course with an increasing tendency towards the end of the experiment (Appendix A, Table A4). An increase in the THI tended to be accompanied by an increase in water consumption over the entire duration of the experiment (Figure 1).

**Figure A4:**
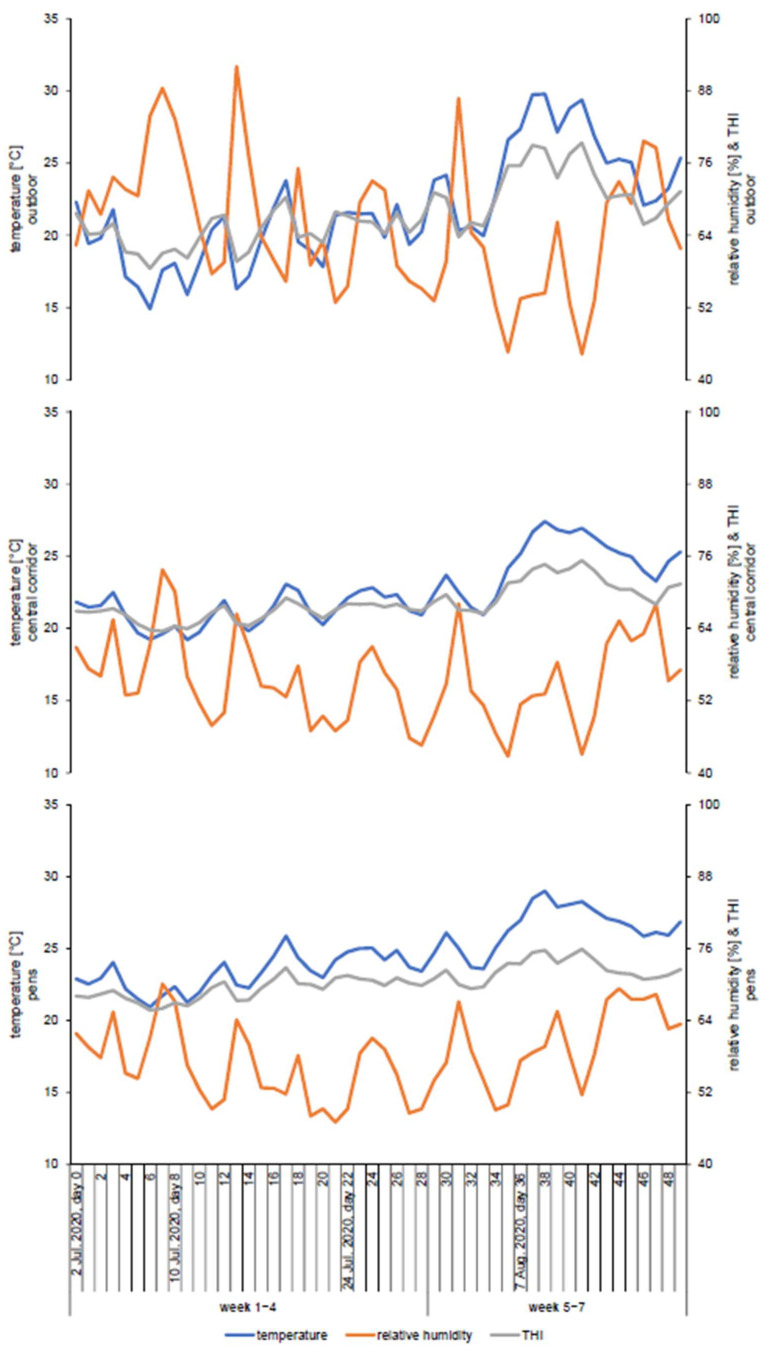
Development of the parameters temperature, relative humidity and THI in the outdoor area, in the central corridor and in the pens during the experiment (daily mean values).

**Figure A5:**
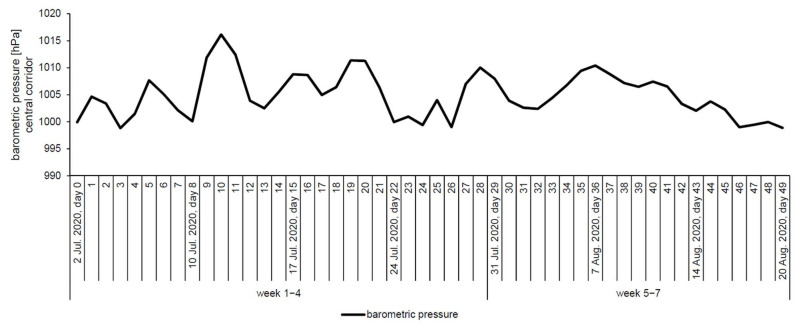
Development of the barometric pressure in the central corridor during the experiment (daily mean values).

Although there were relatively strong temperature fluctuations outside, the temperature in the animal areas was constant. The relative humidity was slightly lower in the animal area than in the outdoor area. The fluctuations in the THI in the animal area were also very small (Appendix A, Figure A4 and Figure A5).

The evaluation scheme of Eigenberg et al. [17], which was also applied by Wegner [10], was used to evaluate THI values. It was noticeable that around 80% of the values measured in the animal area and outdoors and 93% of the values measured in the central corridor were in the normal range (Table 4). It should hereby be noted that the evaluation scheme of Eigenberg et al. [17] had been created for cattle.

**Figure A6:**
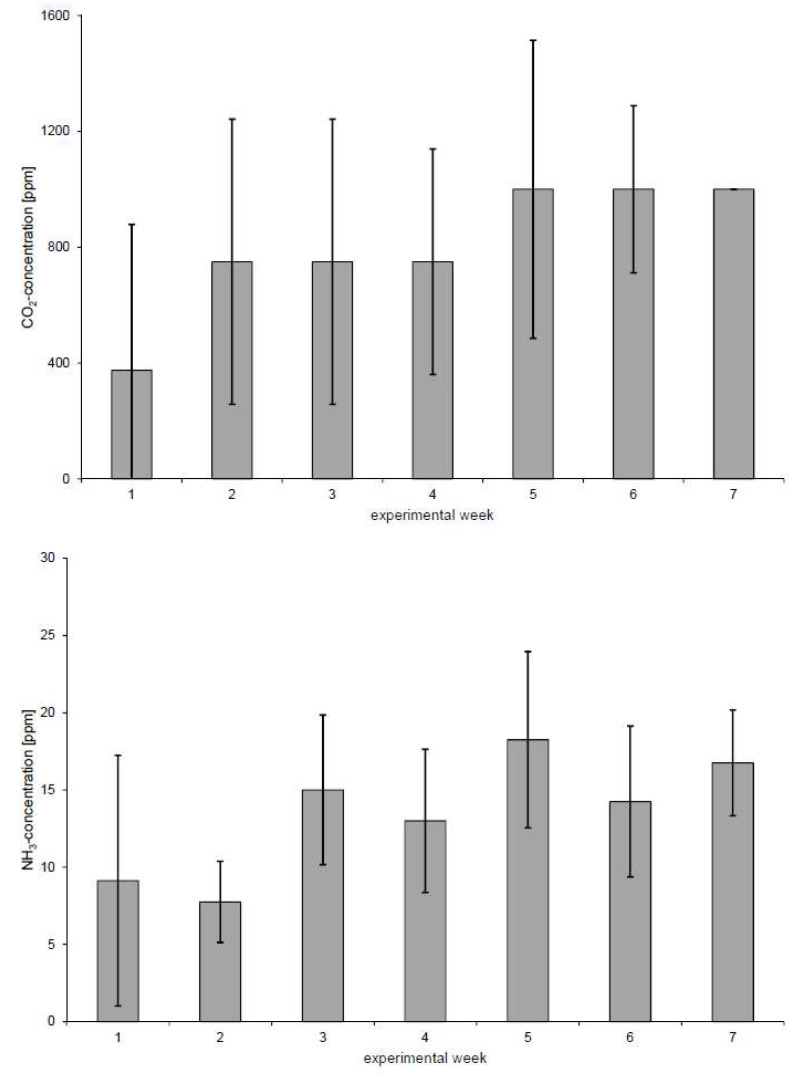
Noxious gas concentrations (mean ± standard deviation) in the animal area (n = 12 measured values/measurement, week 1 with two measurements, week 2–7 with one measurement/week).

The CO_2_ concentrations were below the legal limit [11]. The fluctuations of the values were relatively high. However, it should be considered that the measuring device had a resolution of 1000 ppm, so high fluctuations occurred due to the low resolution alone. The NH_3_ content occasionally exceeded the legal limit [11]. H_2_S could not be detected in any of the measurements (Appendix A, Figure A6).

**Figure A7:**
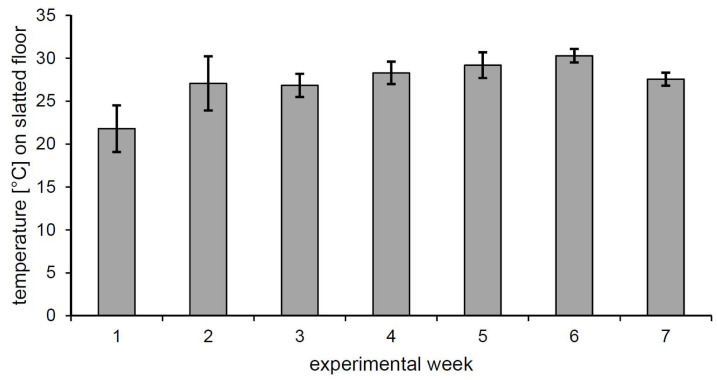
Surface temperature (mean ± standard deviation) of the slatted floor (n = 12 measured values/measurement, week 1 with two measurements, week 2–7 with one measurement/week).

The surface temperature of the slatted floor showed a slightly increasing trend with a maximum in week 6, which was comparable to the temperature development in the compartments (Appendix A, Figure A7).

### 3.1. Water Consumption

**Figure 2:**
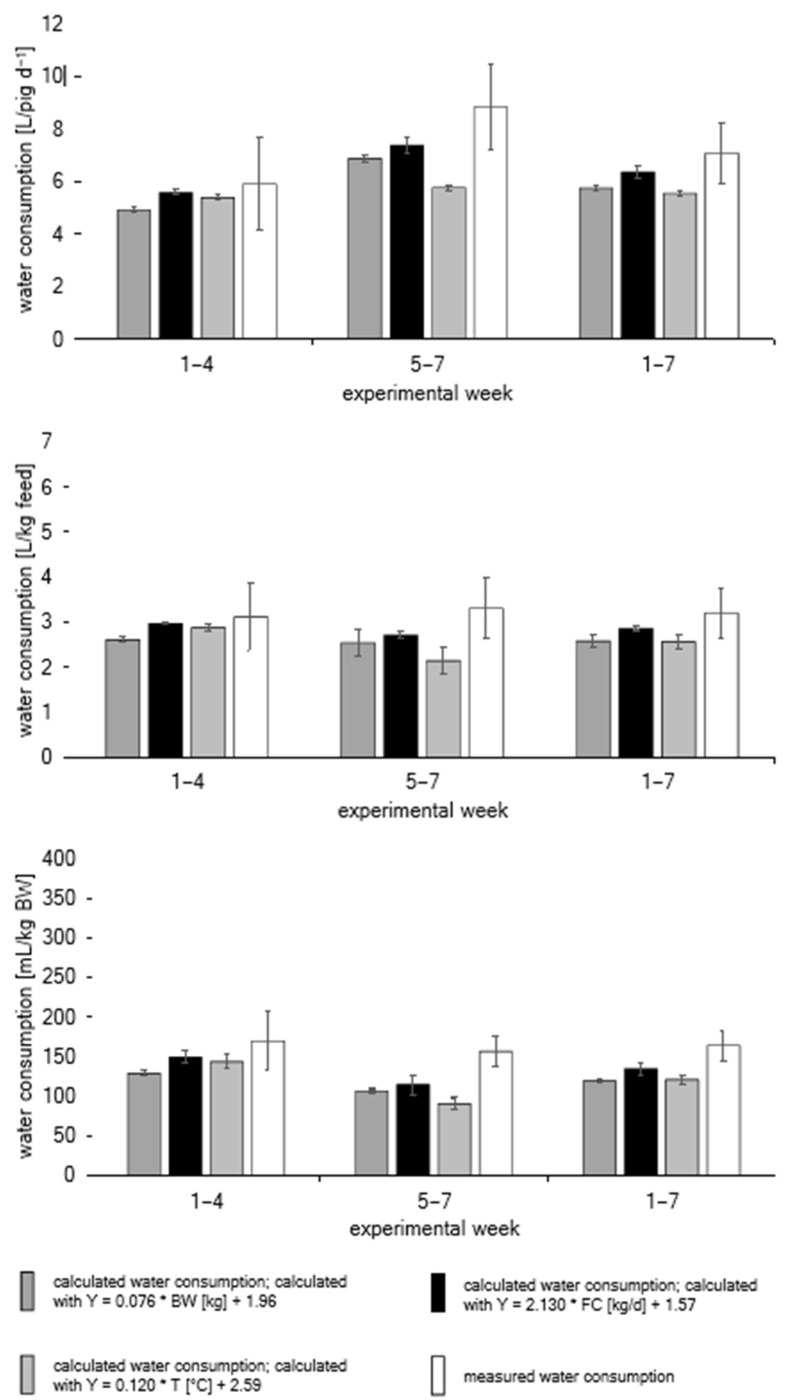
Calculated and measured water consumption per day, related to feed consumption and BW (mean ± standard deviation) according to Equations (2)–(4) [12]. BW = body weight, FC = feed consumption, T = temperature.

The measured water consumption showed an increasing discrepancy between the periods of weeks 1–4 and weeks 5–7 and was compared to the calculated water consumption according to Equations (2)–(4) [12] where the three variables were BW, feed consumption and temperature. Significant differences were found between the calculated and measured water consumption in weeks 5–7 and over the entire fattening phase when comparing the calculated water consumption based on temperature with the measured water consumption. In weeks 5–7, there was also a very high discrepancy of 53% between the measured and calculated water consumption. Water consumption in relation to feed consumption showed a very similar picture, whereby over the entire fattening phase the calculated water consumption was also significantly lower. Significant differences between the calculated water consumption by weight and temperature in weeks 1–4 and weeks 5–7 could be determined for the water consumption in relation to the BW. The largest percentage deviation between the measured and calculated water consumption was found at 72%. Over the whole fattening phase, the calculated water consumption was significantly lower (Figure 2, Table 5).

**Figure 3:**
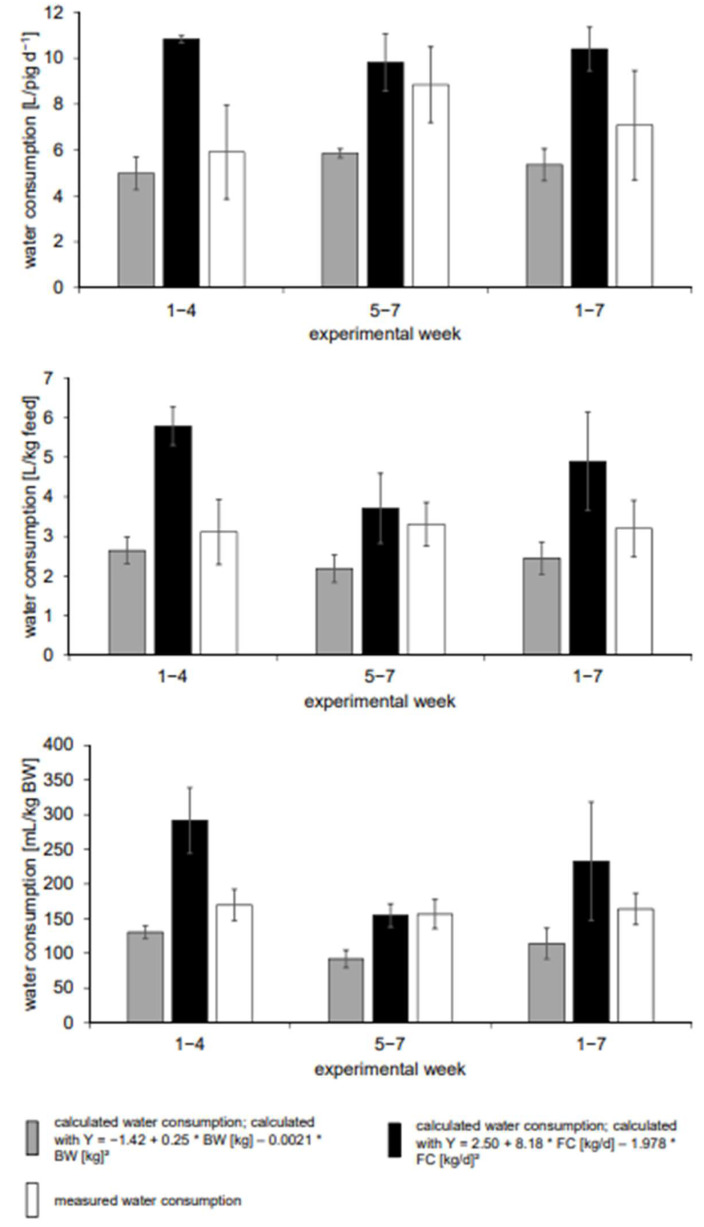
Calculated and measured water consumption per day, related to feed consumption and BW (mean ± standard deviation) according to Equations (5) and (6) [13]. BW = body weight, FC = feed consumption, T = temperature.

When calculating the water consumption values using Equations (5) and (6) [13], the calculation L/pig and day showed that there was a significantly higher calculated water consumption over feed consumption in weeks 1–4 compared to the measured values. In weeks 5–7, on the other hand, the measured water consumption was significantly higher than the calculated water consumption based on the BW. Over the entire fattening period, both calculated values were significantly higher and lower, respectively, compared to the measured water consumption. The results for water consumption per kg of feed showed the same significant differences as the calculation of water consumption per pig and day. In both phases (weeks 1–4 and 5–7) and over the entire fattening phase, the measured water consumption in relation to the BW was always significantly higher than the water consumption calculated based on the BW. In weeks 5–7, there was a percentage deviation of 71% (Figure 3, Table 5).

Overall, the percentage deviations increased from weeks 1–4 to weeks 5–7, except in the calculation of water consumption related to feed consumption by using Equation (6) [13]. Here, the percentage deviation decreased significantly from weeks 1–4 to weeks 5–7. Additionally, an overestimation of water consumption only occurred when using Equation (6) at the peak 46%, while an underestimation occurred in all other calculated values.

**Figure 4:**
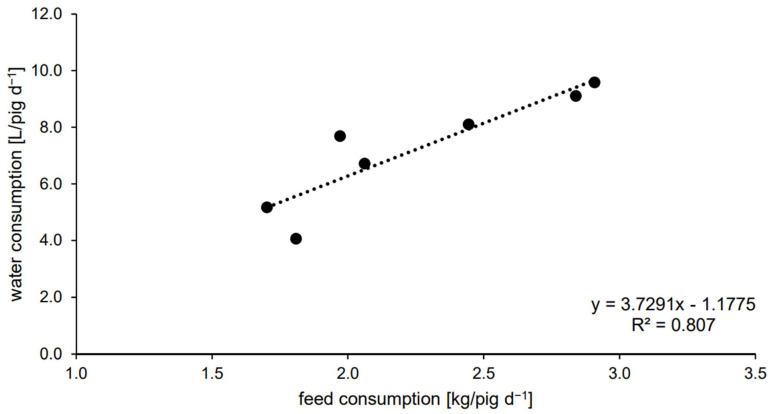
Regression between the measured water consumption and feed consumption (both parameters with weekly mean values of the 4 pens); Pearson correlation coefficient *p*-value = 0.006; n = 4 pens.

In addition to the comparison of the calculated and measured water consumption, the measured water consumption was also correlated with the feed consumption. There was a significant correlation between the two parameters (Figure 4).

### 3.2. Newly Derived Water Consumption Equations

Due to the large deviations between the measured and calculated water consumption, new water consumption equations were derived. As in the literature, the variables BW, feed consumption and temperature were used. In addition, the variable THI, calculated using the equation of the NWSCR [16], was employed as a further variable. In addition to deriving the equations on the basis of the individual variables mentioned, an equation was also deduced including BW and temperature, as well as BW together with THI. Furthermore, the results of the Spearman correlation coefficient and the results of the regression analysis are shown in Table 6.

The Spearman correlation coefficient showed a highly significant correlation between the measured water consumption and the respective predictor variables for all six derived equations. All equations showed high adjusted r-square values in the linear regression model. The best values of 97.95% and 97.94% were found using Equations (4) and (5) with BW and temperature and BW and THI. This was followed relatively closely by Equation (1) with the predictor variable BW, the value of which was 97.73% (Table 6). In Equations (4) and (5), it should be noted that the predictor variables temperature and THI showed a tendency towards significance, and thus the improvement in precision was only slightly missed.

## 4. Discussion

In the course of the evaluation, it was found that water consumption was significantly underestimated using Equations (2)–(4) [12], and that Equations (5) and (6) [13] also partly resulted in an underestimation of water consumption. A possible explanation for this is that the data of Gill [13] are more than 30 years old and the data of Schiavon and Emmans [12] are around 50 years old. In the meantime, an enormous breeding development of pigs has taken place. In recent decades, among other things, daily gains and lean meat content have been increased and feed efficiency improved [20]. As a result, today’s high-performing pigs have a very different genetic makeup and a much higher performance potential than the pigs used in the mentioned studies of Schiavon and Emmans [12] and Gill [13]. Accordingly, in this experiment, high-performing crossbred pigs were used, whereas the data of Schiavon and Emmans [12] were obtained from pigs of the Large White race.

Today’s high-performing pig breeds possess a higher protein content and a lower fat content compared to earlier ones. Since protein implies a higher retention of water than fat, today’s pig breeds already have higher water requirements from a purely physiological point of view. This means that the water requirements of today’s pigs are quite different from those of 30–50 years ago due to both the higher performance potential and the changed body composition [21,22,23].

This shows that the old equations are no longer recommended, as not only genetics but also husbandry conditions have changed. A clear influence of the housing environment in the form of extremely high noxious gases or extreme climatic conditions on the water consumption of the pigs can be excluded. The results of the assessment of the animals on the basis of the animal welfare parameters also showed rather low abnormalities. For example, high ammonia concentrations could lead to tail biting and thus greatly reduce the welfare of the animals [24,25]. However, this was not the case here. Especially towards the end of the experiment, there was a clear increase in the parameter manure on the body, which can be explained by the temperature increase in the same period [26]. Therefore, it can be stated that the husbandry conditions corresponded to the usual standard (Appendix A).

The measured water consumption per kg feed consumption in this study was 3.2 L/kg feed over the entire fattening period. This value was thus in a range that could also be determined in other studies. The following data can be found in the literature:

- 3.43 L/kg dry matter for ad libitum feeding [27];
- 2 to 4 L/kg dry matter intake, on average 3 L/kg dry matter intake [28];
- 2.43 L/kg (growing phase) and 2.13 L/kg (finishing phase) for dry ad libitum feeding [29];
- 2.90 L/kg, respectively 3.10 L/kg [30];
- 2.05 to 5.43 L/kg feed, on average 3.17 L/kg [12];
- 2.91 to 3.64 L/kg feed [13];
- 2.1 to 5.0 L/kg feed for ad libitum feeding [31];
- 2.05 to 5.22 L/kg feed [32];
- 2.10 to 5.43 L/kg feed for ad libitum feeding [4];
- 2.33 to 5.35 L/kg [33].

The water consumption in this study, calculated using Equations (2)–(4) [12], was on average 2.58 L/kg feed consumption (calculation based on BW), 2.86 L/kg feed consumption (calculation based on feed consumption) and 2.57 L/kg feed consumption (calculation based on temperature), which is in the lower range of the literature references. When calculating the water consumption according to Equations (5) and (6) [13], the values of 2.45 L/kg feed consumption (calculation based on weight) and 4.90 L/kg feed consumption (calculation based on feed consumption) could be determined.

The measured water consumption per kg BW was just under 164 mL for the entire fattening period and thus in the upper range of the literature references. The following data can be found in the literature:

- 77.9 mL/kg BW (growing phase) and 74.9 mL/kg BW (finishing phase) [29];
- 112 to 130 mL/kg BW [13];
- 101 to 168 mL/kg BW [31];
- 85 to 234 mL/kg BW [32];
- 63 to 184 mL/kg BW [33].

The water consumption in this study, calculated using Equations (2)–(4) [12], was on average 119.29 mL/kg BW (calculation based on BW), 134.41 mL/kg BW (calculation based on feed consumption) and 120.82 mL/kg BW (calculation based on temperature), which is in the lower range of the literature references. When calculating the water consumption according to Equations (5) and (6) [13], the values of 113.78 mL/kg BW (calculation based on weight) and 232.80 mL/kg BW (calculation based on feed intake) could be determined.

Based on the partly clear deviations between the calculated and the measured water consumption, new water consumption equations were derived. For further validation of these newly established water consumption equations, more data would be very helpful. Values for animals weighing up to approximately 120 kg would be very helpful in order to be able to represent the entire fattening period with the equations. In addition, further data from the other seasons would be very useful in order to also take these into account in the equations. Depending on how much water consumption changes during the seasons, it could also make sense to set up separate equations for summer and winter, as well as another equation for spring and autumn. This would make the actual water consumption of the pigs even more precise.

The six newly derived water consumption equations showed that Equations (4) and (5) in Table 6 with BW and temperature and BW and THI as predictor variables have to be preferred. In the Spearman correlation, both predictor variables were able to achieve the best values with a rounded r_s_ of 0.89 with the predictor variable temperature at 0.87. Equations (4) and (5) also achieved the best values for the RMSE and the adjusted r-square. In terms of practicality, these equations are applicable. Equation (5), however, had an additional benefit compared to Equation (4) in that more climatic parameters were included in the calculation of the water consumption due to the temperature and relative humidity for calculating the THI. It should be noted that only the weekly mean values for the climate data could be used for the derivation of the equations. This meant that the entire variation could not be represented in the derivation.

If temperature or THI are not included as predictor variables in the equation and only the predictor variable BW is used, good results are also obtained. However, the RMSE increases slightly, which indicates a poorer precision of the equation. The adjusted r-square, on the other hand, remains unchanged in Equations (4) and (5) with the climate parameters as additional predictor variables. It can be assumed that this is due to the small database. With a larger database, it is to be expected that the adjusted r-square would become worse in Equation (1) due to the absence of the additional predictor variables temperature and THI. Huynh et al. [34] determined that there was a significant effect on daily weight gain when relative humidity was combined with high ambient temperatures. Furthermore, high temperatures led to a decrease in feed intake, and thus daily weight gains decreased [34,35]. Regarding the use of the variable BW to derive water consumption, this shows that Equation (1) gives good results. The barn climate, however, also has an important influence on animal behaviour. This influence should not be underestimated. Modern ventilation computers can record the temperature and increasingly also the relative humidity. Therefore, a calculation of the THI should cause only minor adjustments in the software. Large group housing with weighing systems can definitely deliver the values of animal weights. However, in the case of small groups, the recording of animal weights is more problematic since the weight of the animals can only be recorded manually by the farmer by weighing individual pigs or certain groups of animals. Due to the lack of automation in this case, the farmer can control the water consumption with the help of the equations if necessary. If it is not possible to record the THI for technical reasons, Equation (1) in Table 6 with the predictor variable BW should be used. This equation also has the third-best statistical parameters. However, further validation of the equations requires more data.

Advanced development of the use of the THI in the pig sector would also be very helpful in order to improve the barn climate design or improve the derivation of water consumption on the basis of the THI. Since pigs can only sweat a little, they regulate their body temperature via the mucous membranes in their mouths in the form of panting [5,6]. The lower the humidity, the easier this works, or the higher the relative humidity, the worse this works [7,8]. Above all, it would be very important to review the classification of THI levels by Eigenberg et al. [17]. The authors, however, established the classifications for cattle. By applying the classification for pigs, Wegner [10] was able to determine that more than 80% of the THI values were in a normal range, but Wegner [10] considered this unlikely and assumed a THI of 66 as the threshold for heat stress in pigs and a temperature of 22 °C at 80% relative humidity as the upper limit of the comfort zone in lactating sows [36]. In addition, it must be considered that the upper critical temperature decreases with increasing relative humidity [34]. However, an increase in temperature has a greater influence on pigs than an increase in relative humidity [34,37]. Which THI values are optimal for fattening pigs must be clarified in further studies. Tests under controlled climatic conditions in climatic chambers would be necessary to simulate different combinations of temperature and humidity, investigate the reaction of the pigs, and finally derive limit values for a THI classification.

To further simplify matters for the farmer, it would be useful if both the water consumption and the climate data could be merged in a barn management software, where the data could be used to create prognoses for the farmer. In addition, the data could be used in a software for early detection of deviations in order to identify possible changes in animal health at an early stage.

## 5. Conclusions

The derivation of new water consumption equations was necessary due to the partly clear differences between the measured and the calculated water consumption. More data is needed to further validate the newly established equations. There is also a great need for further research in the field of barn climate in order to adjust the calculation of the THI and especially its interpretation of heat stress in fattening pigs.

## Figures and Tables

**Table 1:** Animal welfare parameters assessed in the experiment (adapted from Schrader et al. [14]).

Parameter	Level	Definition
tail lesions	0	tail without clearly visible bleeding wound, scab or swelling
	1	tail with clearly visible bleeding wound, scab or swelling
ear lesions	0	ear without clearly visible bleeding wounds and scabs
	1	clearly visible sores and crusts on the ear (especially occurring at the tip, rim or base of the ear)
skin lesions (except tail and ears)	0	<4 line-shaped lesions with ≥5 cm length and no circular lesion with diameter ≥2.5 cm (EUR 2 coin)
	1	≥4 line-shaped lesions with ≥5 cm length or one flat lesion with a diameter ≥2.5 cm (EUR 2 coin)
manure on the body	0	“unsoiled”: <10% of the surface with faecal deposits
	1	“soiled”: ≥10% of the surface with faecal deposits

**Table 2:** Ingredients and analyzed nutrients of the feed in weeks 1–4 and weeks 5–7.

**Ingredients [g/kg]**	**Week 1–4**	**Week 5–7**
barley	32.96	34.71
wheat	38.76	38.12
soybean meal	23.79	22.69
soya oil	1.50	1.50
premix	2.99	2.98
**Nutrient [g/kg dry matter]**	**Week 1–4**	**Week 5–7**
dry matter [g/kg]	901.0	894.7
crude protein	214.3	205.3
crude fibre	42.5	39.4
crude fat	46.0	37.6
starch	452.2	464.0
sugar	39.0	38.8
energy [MJ ME/kg]	13.8	13.7

ME content calculated according to Communications of the Committee for Requirement Standards of the Society of Nutrition Physiology [15]. The feed ration was calculated with Zielwert-Futter-Optimierung (Zifo2) (Bayerische Landesanstalt für Landwirtschaft, Institut für Tierernährung und Futterwirtschaft, Poing/Germany) and analyzed using Weender analysis. Ingredients of the premix: Vitamin A (3a672a) 800000 IE, Vitamin D3 (3a671) 100000 IE, Vitamin E (3a700) 5000 IE, Vitamin B1 100 mg/kg, Vitamin B2 (3a825i) 300 mg/kg, Vitamin B6 200 mg/kg, Vitamin B12 2000 mg/kg, Vitamin K3 (MNB) (3a711) 200 mg/kg, Vitamin C 5000 mg/kg, Biotin 7500 mg/kg, Niacinamide 1250 mg/kg, Pantothenic acid 750 mg/kg, Folic acid 75 mg/kg, Choline chloride 12,500 mg/kg, Iron-Fe from Iron (II)-sulphate-monohydrate (3b103) 7500 mg/kg, Manganese-Mn from Manganese(II)-oxide (3b502) 4000 mg/kg, Zinc-Zn from Zincoxide (3b603) 5000 mg/kg, Iodine-J from Calcium iodate (anhydrous) (3b202) 100 mg/kg and Selenium-Se from Sodium selenite (3b801) 20 mg/kg.

**Table 3:** Performance parameters. Data are presented as mean ± standard deviation.

Phase	Week 1–4	n ^1^	Week 5–7	n ^1^	All	n ^1^
initial BW [kg]	24.3 ± 4.3	79	49.7 ± 6.9	79	24.3 ± 4.3	79
final BW [kg]	49.7 ± 6.9	79	72.5 ± 8.8	73	72.5 ± 8.8	73
daily BW gains [g/d]	906 ± 266	316	1067 ± 351	227	973 ± 314	543
feed consumption [kg/pig d]	1.9 ± 0.2	16	2.8 ± 0.4	12	2.3 ± 0.5	28
feed conversion ratio [kg/kg]	2.2 ± 0.5	16	2.7 ± 0.5	12	2.4 ± 0.5	28
water consumption [kg/pig d]	5.9 ± 2.1	112	8.8 ± 1.7	74	7.1 ± 2.4	186

^1^ measured values; n = 79 pigs at the start and 73 pigs at the end in 4 pens over 7 weeks.

**Table 4:** Percentage distribution of THI values based on the evaluation scheme of Eigenberg et al. [17].

Classification THI *	Pen 1 [%]	Pen 2 [%]	Pen 3 [%]	Pen 4 [%]	Mean Pen 1–4 [%]	Central Corridor [%]	Outside [%]
n ^1^	71,397	71,400	71,250	71,400	4	71,400	71,400
THI ≤ 74 Normal	84	79	88	85	84	93	81
THI > 74–≤ 79 Attention	14	17	11	14	14	6	8
THI > 79–≤ 84 Danger	2	3	1	1	2	1	5
THI > 84 Emergency	0	0	0	0	0	0	7

^1^ measured values; * THI values calculated using the equation of NWSCR [16].

**Table 5:** Statistical analysis between the measured and the calculated water consumption is displayed in Figure 2 and Figure 3.

Unit	L/Pig and Day			L/kg Feed			mL/kg BW		
Phase	Week 1–4	Week 5–7	All	Week 1–4	Week 5–7	All	Week 1–4	Week 5–7	All
n [values]	16	12	28	16	12	28	16	12	28
	adjusted *p*-values								
water consumption	measured								
calculated according to BW ^1^	0.150	0.455	0.087	0.094	0.159	**0.003**	**0.005**	**0.017**	**<0.001**
calculated according to FC ^1^	1.000	1.000	1.000	1.000	1.000	1.000	1.000	0.159	**0.020**
calculated according to T ^1^	1.000	**<0.001**	**0.007**	1.000	**<0.001**	**0.006**	**0.308**	**<0.001**	**<0.001**
calculated according to BW ^2^	0.464	**<0.001**	**0.005**	0.242	**<0.001**	**<0.001**	**0.009**	**<0.001**	**<0.001**
calculated according to FC ^2^	**0.004**	1.000	**<0.001**	**0.005**	1.000	**0.034**	0.115	1.000	1.000

^1^ = calculated with Equations (2)–(4) [12]; ^2^ = calculated with Equations (5) and (6) [13]; significant differences calculated using the Mann–Whitney U-test and the Dunn’s test [19]; BW = body weight [kg/pig]; FC = feed consumption [kg/pig and day]; T = temperature [°C]; *p* < 0.05.

**Table 6:** Results of the Spearman correlation and the regression analysis for the six new equations (y = l/pig d^−1^).

Equation	1	2	3	4	5	6
**Spearman correlation, n = 89,296**						
r_s_	0.90	0.89	0.87	BW: 0.90 T: 0.87	BW: 0.90 THI: 0.89	0.80
*p*-value	<0.0001	<0.0001	<0.0001	BW: <0.0001 T: <0.0001	BW: <0.0001 THI: <0.0001	<0.0001
**Spearman correlation, n = 28**						
r_s_	0.90	0.89	0.87	BW: 0.89 T: 0.87	BW: 0.89 THI: 0.89	0.81
*p*-value	<0.0001	<0.0001	<0.0001	BW: <0.0001 T: <0.0001	BW: <0.0001 THI: <0.0001	<0.0001
**Regression analysis, n = 28**						
regression model	y = 0.142 * BW [kg]	y = 0.103 * THI	y = 0.295 * T [°C]	y = 0.109 * BW [kg] + 0.072 * T [°C]	y = 0.114 * BW [kg] + 0.021 * THI	y = 3.190 * FC [kg/pig d]
f-value (*p*)	1209.19 (<0.0001)	403.36 (<0.0001)	544.69 (<0.0001)	670.39 (<0.0001)	666.31 (<0.0001)	715.42 (<0.0001)
Root Mean Square Error (RMSE) [l/pig d^−1^]	1.13	1.91	1.66	1.07	1.07	1.45
adjusted r-square	0.98	0.93	0.95	0.98	0.98	0.96
coefficient variable t-value (*p*)	34.77 (< 0.0001)	20.08 (<0.0001)	23.34 (<0.0001)	BW: 6.21 (<0.0001) T: 1.96 (0.0605)	BW: 7.70 (<0.0001) THI: 1.92 (0.0663)	26.75 (<0.0001)
Range of application of the new equations: BW: 27.2–75.7 kg/pig; FC: 1.5–3.6 kg/pig d; T: 22.2–28.3 °C; THI: 67.1–75.7						

BW = body weight [kg/pig]; FC = feed consumption [kg/pig d^−1^]; T = temperature [°C]; THI = temperature-humidity index (calculated according to the equation of NWSCR [16]).

## Data Availability

Data are available in a publicly accessible repository. The data presented in this study are openly available in OpenAgrar at DOI 10.3220/Data20230503140839-0 or under the following link: https://www.openagrar.de/receive/openagrar_mods_00085924 (accessed on 3 May 2023).

## Appendix A

**Table A1:** Ventilation settings for exhaust air, supply air and circulating air during the experiment.

Day	1	7	25	46	100	150
**exhaust air**						
temperature curve						
initial temperature [°C]	25.0	21.0	19.0	17.0	15.0	15.0
heating [°C]	24.0	20.0	18.0	16.0	14.0	14.0
ventilation curve						
regulating range [K *]	1.0	1.0	1.0	1.0	1.0	1.0
minimum [%]	5	5	5	5	5	5
maximum [%]	80	80	80	80	80	80
**supply air**						
temperature curve						
initial temperature [°C]	24.0	19.0	17.0	15.0	13.0	13.0
heating [°C]	23.0	18.0	16.0	14.0	12.0	12.0
ventilation curve						
regulating range [K *]	1.0	1.0	1.0	1.0	1.0	1.0
minimum [%]	10	10	10	10	10	10
maximum [%]	70	70	70	70	70	70
**circulating air**						
temperature curve						
initial temperature [°C]	25.0	21.0	19.0	17.0	15.0	15.0
heating [°C]	24.0	20.0	18.0	16.0	14.0	14.0
ventilation curve						
regulating range [K *]	1.0	1.0	1.0	1.0	1.0	1.0
minimum [%]	0	0	0	0	0	0
maximum [%]	100	100	100	100	100	100

* K = Kelvin.

Schiavon and Emmans [12] used data from six studies to evaluate their model. The housing conditions in the six studies are shown below (Table A2). In total, the authors excluded two data sets from the six studies for their test data set. In one test data set, the animals were fed less than 20 g/kg body weight, and the other the diet contained 1.1 g sodium chloride/kg feed. A total of 68 data points were included in the test data set. The pigs had a live weight of 17–74 kg and were fed between 0.7–3.51 kg of feed per day. They consumed 2–9 l/water per day [12]. Table A3 describes the experimental conditions at Gill [13].

**Table A2:** Description of the housing conditions of the six studies used by Schiavon and Emmans [12] for their test data set.

References	Morrison and Mount [4]	Mount et al. [31]	Close et al. [32]	Holmes and Mount [33]	Hagsten and Perry [38]	Verstegen et al. [39]
genetic	Large White	Large White	Large White	Large White	no details	Large White
sex	castrated male pigs	selection of litter of origin, sex, weight and growth rate according to Holmes and Mount [33]	as with Holmes and Mount [33]	castrated male pigs balanced for sex and the origin of the pigs in relation to the litter, growth rate and weight	crossbred barrows	castrated male pigs selection of litter of origin, weight and growth rate according to Holmes and Mount [33]
body weight	mean body weight close to 20 kg	21–73 kg per pig	initial mean values between 21.0 and 34.0 kg/pig	initial mean values between 20.3 and 61.5 kg/pig	initial mean values between 12 and 49 kg/pig	initial weight: 24–28 kg/pig end weight: 35–45 kg/pig
feeding	ad libitum, dry feeding	42 g feed/kg body weight per day to maximal intake	feeding twice a day “commercial growers” meal from about 9 weeks of age until slaughter 34 g/kg meal per body weight until up to 52 g/kg meal per body weight in stages	four feeding times “commercial growers” meal from about 9 weeks of age until slaughter 42 g feed/kg body weight per day up to a maximum daily meal of 1.83 kg	ad libitum corn-soy-bean meal with supplement salt	feeding twice a day, dry feeding 45 g feed/kg BW and 52 g feed/kg BW at 8 °C 39 g feed/kg BW and 45 g feed/kg BW at 20 °C commercial grower diet
water	ad libitum	ad libitum	ad libitum 1 L/kg feed added to the feed	outside the feeding time ad libitum 3 L water mixed with the feed per feeding time	in the pens: deionized water ad libitum in the metabolism stall, twice daily	ad libitum
housing	temperature-controlled pen calorimeter *	temperature-controlled pen calorimeter *	temperature-controlled pen calorimeter *	temperature-controlled pen calorimeter *	first pen with 1.22 m × 2.44 m and second pen with 2.44 m × 4.88 m both with concrete floor metabolism stall with 0.35 m × 1.27 m	Calorimeter *
climate	temperature controlled, humidity not different temperature levels between 20 and 33 °C	different temperature levels between 7 and 33 °C	different temperature levels between 7 and 30 °C	different temperature levels between 9 and 30 °C	no details	two temperature levels: 8 and 20 °C

* described by Mount et al. [40]. BW = body weight.

**Table A3:** Description of the housing conditions for deriving the water requirement equations used here from Gill [13].

Reference	Gill [13]
genetic	no details
sex	male and female pigs, per 12 pigs
body weight	start: mean body weight 14.3 kg/pig end: mean body weight 78.2 kg/pig
feeding	single feed hopper in every pen with ad libitum feeding, different levels of potassium (K) between 8–17 g K/kg air-dry feed
water	ad libitum
housing	two performance testing houses with 4 pens
climate	temperature between 14.7 and 19.1 °C

**Table A4:** Prevalence and CS of animal-based welfare parameters and parameter distribution. Data are presented as mean ± standard deviation.

	Experimental Week								Experimental Week						
	1	2	3	4	5	6	7	Unit	1	2	3	4	5	6	7
parameter	prevalence [%]							CS	0.11 ± 0.16	0.15 ± 0.19	0.23 ± 0.21	0.12 ± 0.14	0.26 ± 0.21	0.29 ± 0.19	0.38 ± 0.25
tail lesions	0.00	2.56	1.90	0.00	3.23	5.19	7.38	% of CS	0.00 ± 0.00	0.64 ± 3.96	0.47 ± 3.42	0.00 ± 0.00	0.81 ± 4.43	1.30 ± 5.57	1.85 ± 6.56
ear lesions	18.57	16.03	20.89	1.27	15.48	27.92	44.97		4.64 ± 9.74	4.01 ± 9.20	5.22 ± 10.19	0.32 ± 2.80	3.87 ± 9.07	6.98 ± 11.25	11.24 ± 12.48
skin lesions (except tail and ears)	24.05	12.82	13.92	5.06	15.48	4.55	23.49		6.01 ± 10.71	3.21 ± 8.38	3.48 ± 8.68	1.27 ± 5.50	3.87 ± 9.07	1.14 ± 5.22	5.87 ± 10.63
manure on the body	1.27	28.85	53.80	42.41	71.61	76.62	77.85		0.32 ± 2.80	7.21 ± 11.36	13.45 ± 12.50	10.60 ± 12.39	17.90 ± 11.31	19.16 ± 10.62	19.46 ± 10.42
n [assessment results] ^1^	237	156	158	158	155	154	149		237	156	158	158	155	154	149

CS minimum: 0.00, CS maximum: 1.00. ^1^ two assessments/week, experimental week 1 three assessments.
